# SPANOL (SPectral ANalysis of Lobes): A Spectral Clustering Framework for Individual and Group Parcellation of Cortical Surfaces in Lobes

**DOI:** 10.3389/fnins.2018.00354

**Published:** 2018-05-31

**Authors:** Julien Lefèvre, Antonietta Pepe, Jennifer Muscato, Francois De Guio, Nadine Girard, Guillaume Auzias, David Germanaud

**Affiliations:** ^1^Centre National de la Recherche Scientifique, ENSAM, LSIS, Aix Marseille University, University of Toulon, Marseille, France; ^2^Centre National de la Recherche Scientifique, Institut de Neurosciences de la Timone, Aix Marseille University, Marseille, France; ^3^Institut National de la Santé et de la Recherche Médicale, University Paris Diderot, Sorbonne Paris Cité, UMR-S 1161, Paris, France; ^4^Centre National de la Recherche Scientifique, CRMBM, Aix Marseille University, Marseille, France; ^5^APHM, Hopital de la Timone, Service de Neuroradiologie, Marseille, France; ^6^CEA, Neurospin, UNIACT, Equipe Neuropédiatrie, Gif-sur-Yvette, France; ^7^Institut National de la Santé et de la Recherche Médicale, Université Sorbonne Paris Cité, CEA, UMR 1129, Paris, France; ^8^APHP, Hopital Robert-Debré, DHU Protect, Service de Neurologie Pédiatrique et des Maladies Métaboliques, Université Paris Diderot, Paris, France

**Keywords:** cortical parcellation, spectral clustering, Laplace-Beltrami operator, fetal MRI, group-wise analysis

## Abstract

Understanding the link between structure, function and development in the brain is a key topic in neuroimaging that benefits from the tremendous progress of multi-modal MRI and its computational analysis. It implies, *inter alia*, to be able to parcellate the brain volume or cortical surface into biologically relevant regions. These parcellations may be inferred from existing atlases (e.g., Desikan) or sets of rules, as would do a neuroanatomist for lobes, but also directly driven from the data (e.g., functional or structural connectivity) with minimum a priori. In the present work, we aimed at using the intrinsic geometric information contained in the eigenfunctions of Laplace-Beltrami Operator to obtain parcellations of the cortical surface based only on its description by triangular meshes. We proposed a framework adapted from spectral clustering, which is general in scope and suitable for the co-parcellation of a group of subjects. We applied it to a dataset of 62 adults, optimized it and revealed a striking agreement between parcels produced by this unsupervised clustering and Freesurfer lobes (Desikan atlas), which cannot be explained by chance. Constituting the first reported attempt of spectral-based fully unsupervised segmentation of neuroanatomical regions such as lobes, spectral analysis of lobes (Spanol) could conveniently be fitted into a multimodal pipeline to ease, optimize or speed-up lobar or sub-lobar segmentation. In addition, we showed promising results of Spanol on smoother brains and notably on a dataset of 15 fetuses, with an interest for both the understanding of cortical ontogeny and the applicative field of perinatal computational neuroanatomy.

## 1. Introduction

The existence of brain regions associated to specific functions (Friston, 2002) as a dominant paradigm in neuroanatomy is a legacy from the XIXth century. This localizationist view has proved more complex and several concurrent segmentations of the brain have been proposed based on microarchitecture first (Zilles and Amunts, 2010), then on neuroimaging studies, in particular with MRI in the past 20 years. *Brain parcellations* have received considerable interest not only because of their deemed underlying biological reality but also because of their advantages in fMRI studies, their ability to reduce the high dimensionality of the data, and their use in object-based strategies to overcome the shortcomings of spatial normalization (Thirion et al., 2006). Data-driven MRI based parcellations are most frequently sought, for instance with fMRI data (Thirion et al., 2014) or anatomical connectivity (Lefranc et al., 2016; Parisot et al., 2016) or both (Glasser et al., 2016). Yet, brain parcellations based only on the anatomy have been mostly built from information provided by experts and manual labeling. For instance the information of manually delineated cortical sulci has been used to provide volumic (Lohmann and von Cramon, 2000; Tzourio-Mazoyer et al., 2002) or surfacic regions (Cachia et al., 2003; Klein and Tourville, 2012), with different strategies to transfer the labeled information by using registration toward an atlas (Tzourio-Mazoyer et al., 2002) or by working more at the level of the single subject (Cachia et al., 2003; Klein and Tourville, 2012). Even model-based parcellations, like the one proposed in Auzias et al. (2016), require a preliminary labeling of some cortical sulci at the subject level. There are, however, recent attempts of *unsupervised* parcellations procedures, using spectral analysis (Germanaud et al., 2012) or 'sulcal pits' concepts (Auzias et al., 2015).

A full unsupervised segmentation of the cortical surface into anatomically meaningful entities remains a very challenging task and there might even be no a priori reasons to hope to obtain one, at least from a non-comprehensive input dataset. Nevertheless preliminary results of two previous studies of our group (Lefevre et al., 2014; Pepe et al., 2015) suggested an intriguing relationships between parcellations obtained by spectral clustering and what has been known for a long time as *brain lobes* (Gratiolet, 1854). Brain lobes are a coarse but well-accepted anatomo-functional segmentation of the brain into 5 parts: frontal, parietal, temporal, occipital and insular. This segmentation is not absolute since for instance a 6th limbic part is sometimes proposed. Besides, if unambiguous landmarks ground some lobar limits (e.g., central sulcus as frontal-parietal lateral limit or parieto-occipital sulcus as a parietal-occipital internal limit), others are rather ill-defined (e.g., in the occipital-temporal continuum).

Spectral clustering has been largely studied, both theoretically and empirically, mostly in the machine learning community (Ng et al., 2002; Von Luxburg, 2007) but also in computer graphics for mesh segmentation purposes (Liu and Zhang, 2004). Spectral clustering requires a laplacian matrix (in the discrete or graph settings) which corresponds to the Laplace-Beltrami Operator (L.B.O.), often considered as the “swiss army knife” of the geometry processing. Recently this mathematical operator has been more and more popular in the field of neuroimaging, with applications to the description of gyrification pattern (Germanaud et al., 2012; Rabiei et al., 2015), to anatomo-functional variability (Lombaert et al., 2015), in shape registration (Lombaert et al., 2013b; Lefèvre and Auzias, 2015), and in shape classification (Lai et al., 2009; Wachinger et al., 2015).

In this article our contributions are threefold:

- We describe a *general framework* to parcellate a group of cortical surfaces in an arbitrary number of connected regions based on a spectral clustering algorithm adapted to triangular meshes. Only the geometry of the brain is considered and a limited supervised information (cingulate pole labeling) can be injected.
- When restricted to 6 regions, the resulting parcellations offer striking *analogies with brain lobes* that can be precisely quantified. The value 6 is optimal regarding a statistical procedure to test the independence of two parcellations.
- We test the robustness of spectral clustering to surface perturbation and in particular to unfolding. Our approach is also tested on fetal cortical meshes, providing unsupervised segmentations of the immature brain that are consistant with the adult stage. Those results suggest that folding pattern itself play a minor role in the definition of spectral lobes, that could be mainly determined by the global shape of the brain.

## 2. Methods

### 2.1. Spectral clustering

Spectral clustering has become a very popular method in machine learning to segment a set of points in different groups based only on pairwise similarities between those points. A dimension reduction step is performed on a matrix derived from the similarities by extracting its *K* first eigenvectors. Then, a classic clustering algorithm such as a *K*-means is performed on these eigenfunctions (Ng et al., 2002; Von Luxburg, 2007).

Graph laplacians are often chosen to define the similarity matrix because of suitable mathematical properties (non-negativity and semi-definiteness) that make them diagonalizable. In our application to cortical surfaces, it is crucial to use a matrix that makes sense in terms of shape and allows fast computations. The Laplace-Beltrami Operator and its discrete representation with Finite Element Methods (F.E.M.) are convenient because they satisfy the required mathematical properties, no parameters tuning is necessary, and the derived matrices are sparse which guarantees efficient computation of eigenvectors thanks to Krylov subspace methods as implemented in ARPACK library. Besides it is interesting to note that, following Belkin and Niyogi (2008), the Laplace-Beltrami Operator can be approximated by a point cloud Laplace Operator which corresponds exactly to an affinity matrix with a Gaussian kernel. Therefore there is an asymptotic equivalence between spectral clustering with Laplace-Beltrami Operator and the traditional one with affinity matrix.

The resulting eigenvectors correspond to an approximation of the *Fourier modes* of the ideal cortical surface. They reflect the intrinsic geometry of the brain shape, independently of how it is embedded in the 3D space. From a more physical point of view, Fourier modes correspond to vibration modes of the surface, following a close analogy to the well-known Chladni plates (Chladni, 1787). Mathematically, Fourier modes correspond to real valued functions ϕ_*i*_ defined on a genus-0 surface S that satisfy:

(1) $$\begin{array}{r} -\Delta_{S}\phi_{i}=\lambda_{i}\phi_{i}. \end{array}$$

where λ_0_ = 0 ≤ λ_1_ ≤ λ_*i*_… are eigenvalues that can be interpreted as spatial frequencies. $\Delta_{S}$ is the Laplace Beltrami Operator of the surface S (see Berger, 2003 for more details).

Visually Fourier modes reveal spatial oscillations whose number increases with *i*. More precisely, the number of connected regions with constant sign (also called *nodal domains*) is bounded between 2 and *i* + 1, because of the celebrated Courant Nodal theorem. Such eigenfunctions/Fourier modes are represented in the **Figure 8**.

On a triangular mesh approximating S with *N* vertices, it is possible to apply the framework of Finite Element Method. A discretized Fourier mode and its corresponding frequency can then be obtained as a vector *U* = (_*u*_*i*_)*i* = 1..*N*_ and a scalar λ such that:

(2) $$\begin{array}{r} \text{G}U=\lambda \text{M}U, \end{array}$$

where the expression of the two matrices, in the case of first order F.E.M., are given in Lefèvre and Mangin (2010).

**Figure 1:**
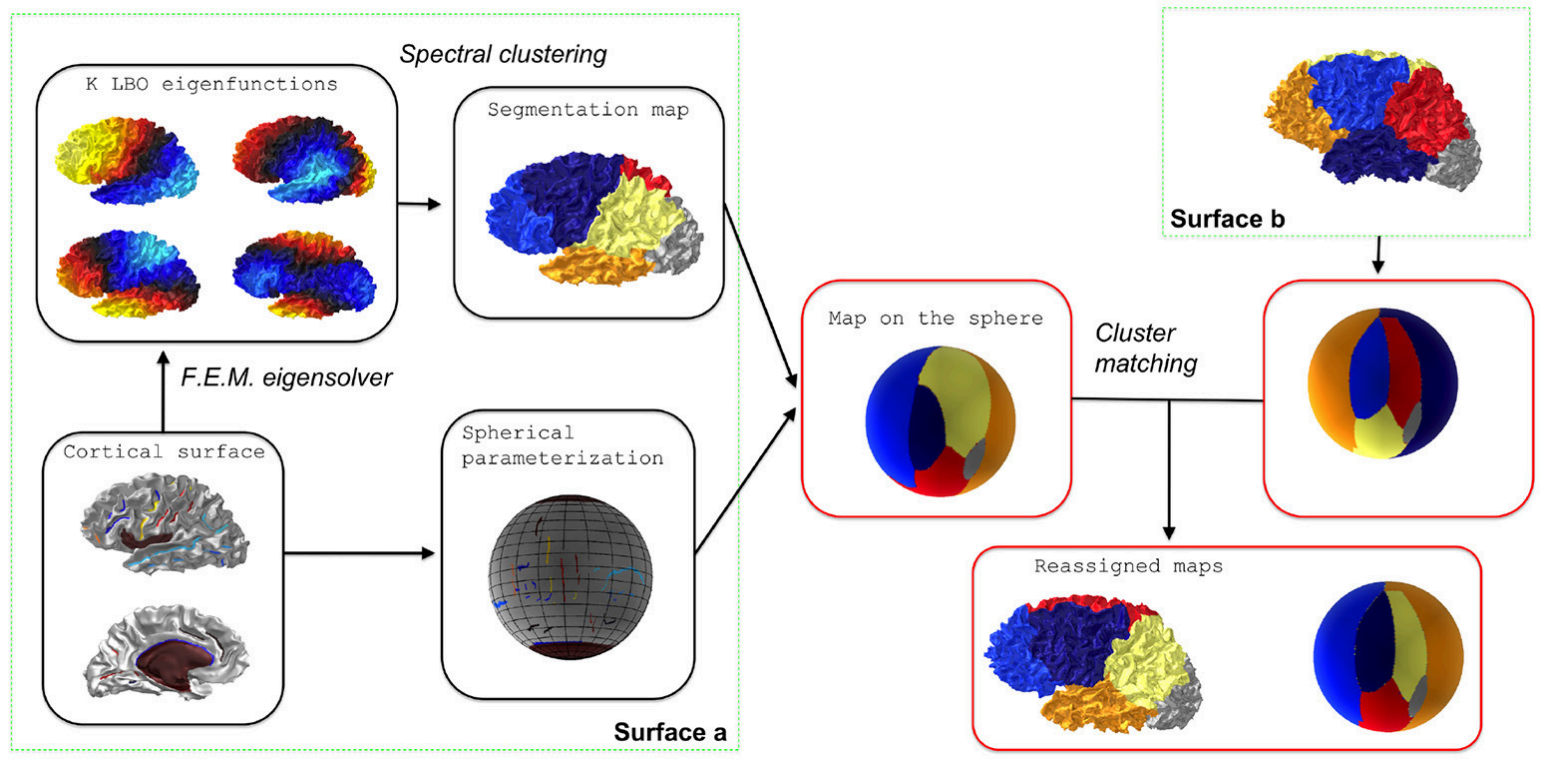
Flowchart of individual spectral clustering and inter-individual strategy for relabeling. The cluster matching step is achieved via the Munkres algorithm.

The two steps of the spectral clustering adapted to the Laplace-Beltrami Operator are summarized on the left block of Figure 1 and in Algorithm 1.

**Algorithm 1:** Cortical surface segmentation

**Require:** integer K, mesh defined by
*n*_*V*_ × 3 array of points V
*n*_*T*_ × 3 array of triangles T
1: Compute sparse matrices G and M of size *n*_*V*_ × *n*_*V*_
2: Find K first eigenvectors X(:,1),…,X(:,K) of GX=λ MX
3: Apply K-means clustering to the *n*_*V*_ × K array X to get an *n*_*V*_ × 1 array labels
4: **return** labels

### 2.2. Specific tunings for spectral clustering of lobes

In the previous part we exposed the general mechanism to apply spectral clustering on general surfaces. We propose here three variations in the context of cortical surface segmentation.

#### 2.2.1. Removing of non-cortical vertices in the clustering

First of all we want to incorporate in the framework some supervised information that would not be efficiently extracted from the data by the spectral analysis. Namely, we consider in our experiments labeled vertices corresponding to the cingular pole, as defined in Auzias et al. (2013), to be excluded from the spectral clustering. This choice is legitimated by the fact that the surface of the cingular pole cannot be considered as homologous to the rest of the mesh, not being cortical, but rather an artificial filling of the hole created by the segmentation procedure when dissociating the two hemispheres. Futhermore, in line with the underlying hypothesis that Fourier modes may be informative for surface parcellation, it would make sense, if the clustering process had trouble dealing with this artificial surface of cingulate pole, to treat it differently.

From a computational point of view we adopt a simple heuristic: (a) we compute first the eigenfunctions of the entire mesh, (b) we exclude the regions for which we have the desired information (e.g., cingular pole), (c) we run the spectral clustering. In the following we will use the term *constrained spectral clustering* when the cingular pole is excluded from the segmentation process, and *unconstrained spectral clustering* in the other case.

#### 2.2.2. Vary the number of clusters and eigenfunctions

We evaluate the qualitative influence of a given Fourier mode on a resulting segmentation. Explicitly, with an anatomical model that includes 6 regions (5 lobes and the cingular pole), the unconstrained clustering requires 6 eigenvectors (including the trivial one) to define 6 clusters and at first sight, the constrained clustering would deal with only 5 eigenvectors to define the 5 unsupervised clusters. Thus, as a test for an add-on effect of a single eigenvector, we will consider only four cases: for the unconstrained approach, the number of eigenvectors is 6 or 7, and 5 or 6 for the constrained clustering.

#### 2.2.3. Effect of smoothing

The sensitivity of the spectral clustering to cortical surface deformations may be an important issue both for theoretical (e.g., which aspect of cortical shape impacts the clustering) and practical (e.g., stability of the segmentation along development or in case of focal lesion) reasons. We tested this sensitivity by deforming the cortical surface and computing again eigenfunctions and spectral clusters on the deformed surface. For general graphs, this issue of relating clustering error to graph perturbation has been addressed in the litterature of machine learning (Huang et al., 2009). But theoretical results are often limited to two clusters and assume a small perturbation. We adopted an empirical approach and explored a family of perturbed surfaces obtained by the mean curvature flow (Huisken, 1984). This geometric flow is able to smooth a folded cortical surface and it has been shown experimentally that it produces no singularities in the case of cortical surfaces (Lefevre et al., 2013). Moreover, this process is able to mimic the backward emergence of cortical folding pattern (Lefevre et al., 2013). Hence, the combination of mean curvature flow with spectral clustering may be seen as a first step of investigation to ensure the stability of a cortical parcellation along the brain development and its dependency to the folding level.

### 2.3. From subject level to group level segmentation

**Figure 2:**
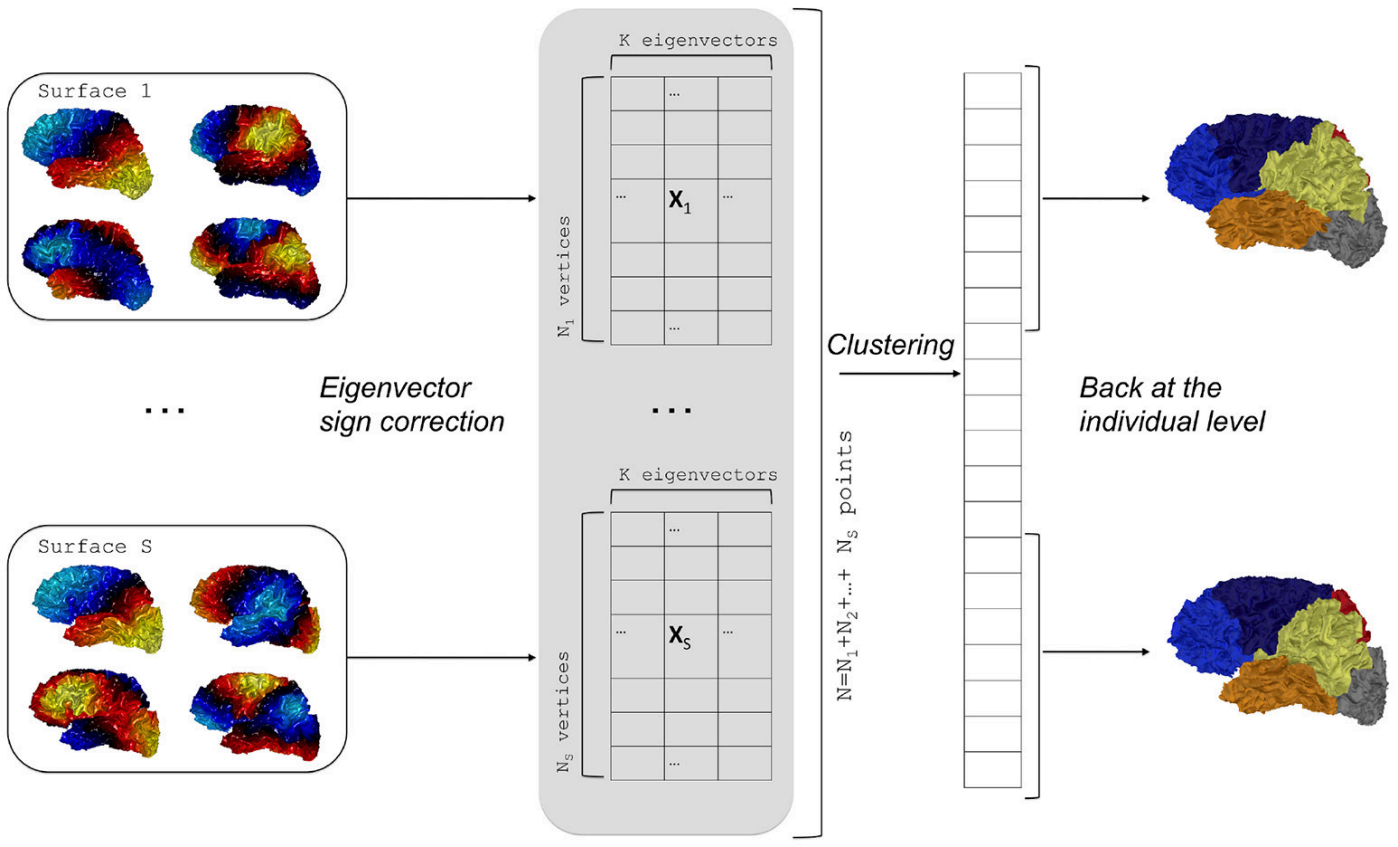
Flowchart of group spectral clustering. Individual eigenvectors are computed for each surface and the sign ambiguity is corrected. Concatenation of eigenvectors yields a large array on which a clustering is performed. Last, the labels are back-projected on the individual surfaces.

When spectral clustering is performed for different surfaces independently there is no guarantee (1) that clusters exhibit a good reproducibility at the group level and, even if segmentation are consistent, (2) that corresponding parcels are assigned to a same label. Starting from our preliminary work (Lefevre et al., 2014) we extended the analysis of point (1) by inspecting extremal parcellations to illustrate the variability. To satisfy point (2) we proposed two different strategies:

- Matching of individual parcellations
  This relabeling strategy is illustrated on the right block of Figure 1. To achieve consistent labelings, the individual segmentations obtained by the spectral clustering are mapped onto a common spherical template 𝕊^2^ where a relabeling is performed. Note that any spherical mapping approach could be suitable if it transforms the global shape of the brain in a consistent way across subjects. Even *ad hoc* methods based on affine registration might work in practice but the choice of using a spherical template is suppported by the evaluation of the reproducibility (see later). In this paper we used the Freesurfer method which controls area distortions (Fischl et al., 1999), a property that will be usefull in section 2.5.
  For each surface *s* we have a mapping $m_{s}:S_{s}\to {𝕊}^{2}$. A segmentation map $f_{s}:S_{s}\to \left\{1,\ldots ,K\right\}$ can then be extended on the spherical template as a composition $g_{s}=f_{s}○m_{s}^{-1}$. Given two surfaces *s* and *s*′ we can obtain a *K* × *K* table whose value at cell (*k, l*) is given by the cardinality of the set $g_{s}^{-1}\left(k\right)\cap g_{s^{\prime }}^{-1}\left(l\right)$. It is simply a quantification of the overlap between two clusters given by labels *k* and *l* for different surfaces.
  Based on the previous table, it is possible to find a relabeling (i.e., a permutation σ_*s*_′:{1, …, *K*} → {1, …, *K*}) that maximizes the degree of overlap between the two segmentations by taking surface *p* as a reference. This step is achieved via the Munkres algorithm that solves the assignment problem Kuhn (1955).
- Group spectral clustering
  Another possibility consists in applying a global clustering to all the individual eigenvectors pooled together.
  Formally, let us consider surfaces $S_{1},\ldots ,S_{S}$ with *N*_1_, …, *N*_*S*_ vertices respectively, while **X**_1_, …**X**_*S*_ are the corresponding arrays of *K* first eigenvectors. We can build a big vector **X** in ℝ^*N* × *K*^, where *N* = *N*_1_ + … + *N*_*S*_, as a concatenation of all the eigenvectors of the *S* surfaces. The *K*-means clustering is applied to the vector **X** which yields a big vector of labels that produces individual segmentations. This step is summarized in Algorithm 2 and in Figure 2.
  Given an eigenfunction Φ associated to an eigenvalue λ, note that −Φ is also an eigenfunction. To overcome this sign ambiguity issue, we used a simple correlation of two eigenfunctions resampled on the common sphere and switched the sign of one eigenfunction if the correlation was negative.

**Algorithm 2:** Group spectral clustering

**Require:** integer K, *S* eigenvectors obtained in Algorithm 1 and
stored in
*S* × 1 cell array EV
1: **Build** X=[EV{1};…;EV{S}] N=[length(EV{1});…;length(EV{S})]
2: Apply K-means clustering to the sum(N) × K array X to get a sum(N) ×1 array group_labels
3: **for** s=1:S **do**
4: Compute i1=sum(N(1:s-1))+1 and i2=sum(N(1:s))
5: Build labels{s}=group_labels(i1:i2)
6: **end for**
7: **return** labels

### 2.4. Evaluation metrics

We propose here two different metrics to evaluate the quality of parcellations across a group of surfaces. The first one assesses the relationship between cluster boundaries and some anatomical landmarks that have been identified by neuroanatomical experts (sulcal lines). The second metric quantifies the distance between spectral clusters and reference clusters (obtained by concatenating smaller parcels obtained with the Desikan atlas in the Freesurfer software Desikan et al., 2006).

#### 2.4.1. Spectral boundaries and landmarks

We consider *M* anatomical landmarks that will be compared to cluster boundaries (*M* = 2 in practice). In particular, Central Sulcus (C.S.) and Parieto-Occipital Sulcus (P.O.S.) are very important landmarks since they are reliable borders between lobes. For each discrete sulcal line $L_{m}$ we can define a mean distance to the boundaries of the segmentation map B of a given subject:

(3) $$\begin{array}{r} D_{m}=\frac{1}{\left|L_{m}\right|}\underset{i\in L_{m}}{\sum }d\left(i,B\right) \end{array}$$

where *d*(., .) is the geodesic distance from a point to a set of points. In practice we use the Fast Marching Algorithm and the Matlab implementation of Peyré and Cohen (2008) to obtain a distance map from B to any vertex of the mesh. The mean distance *D*_*m*_ is also expressed as a percentage of the largest geodesic distance of the cortical mesh as approximated by extremal points of the Fiedler vector (Lefevre et al., 2012).

#### 2.4.2. Comparison of two parcellations

One can compare two segmentations of a mesh by considering them as partitions of the *n* vertices of the template sphere and computing a distance between these partitions. Note that the two parcellation maps may have different number of labels and we will denote them as *K* and *L* in the following.

We have used the *rand distance* which is frequently employed to obtain partition distances or partition similarities (*rand index*). It has the interesting property to be a mathematical distance and to have a very fast computation time. The rand distance is based on the number *a* of pairs of vertices that are in the same cluster in the two partitions, and the number *b* of pairs of vertices that are in different clusters in both partitions. The values of *a* and *b* can be obtained from the contingency table *T*_*k, l*_ between the two segmentations (e.g., following Denœud and Guénoche, 2006):

(4) $$\begin{array}{rcl} a & = & \frac{1}{2}\overset{K}{\underset{k=1}{\sum }}\overset{L}{\underset{l=1}{\sum }}T_{k,l}\left(T_{k,l}-1\right) \end{array}$$

(5) $$\begin{array}{rcl} b & = & \frac{1}{2}\overset{K}{\underset{k=1}{\sum }}\overset{L}{\underset{l=1}{\sum }}\left(n-T_{k,l}\right)\left(n-T_{k,l}-1\right) \end{array}$$

where *T*_*k, l*_ is the number of vertices that have a label *k* (*k* between 1 and *K*) in the first segmentation and a label *l* in the second segmentation (between 1 and *L*).

Then the formula for the rand distance is simply :

(6) $$\begin{array}{r} 1-\frac{a+b}{n\left(n-1\right)/2} \end{array}$$

To evaluate the overlap between each Freesurfer lobe and the corresponding spectral cluster we have chosen *Dice coefficients*, which are commonly used in neuroimaging but do not correspond to mathematical distances. The Dice between two sets *A* and *B* is given by:

(7) $$\begin{array}{r} \frac{2|A\cap B|}{\left|A|+|B\right|}. \end{array}$$

0 corresponds to no overlap, while 1 implies equality of *A* and *B*.

#### 2.4.3. Consensus parcellation

We aim at obtaining consensus maps for visualization purposes and we have used fast and simple methods.

First it is possible to define an intuitive average segmentation in *K* clusters by a *voting strategy*. At each vertex *i* of the sphere we consider the label that is present the most which can also be mathematically written as:

(8) $$F\left(i\right)=\arg\;\underset{k\in \left[1,K\right]}{\max}\left|\left\{s=1..S/g_{s}\left(i\right)=k\right\}\right|$$

### 2.5. Statistical evaluation

We propose a statistical test to determine if a brain parcellation (a mapping *f*_*s*_) obtained by spectral clustering has a statistical relationship with a segmentation map obtained by an expert system, e.g., a Freesurfer parcellation.

For a given subject *s*, an expert system provides a mapping $r_{s}:M_{s}\to \left\{1,\ldots ,L\right\}$. Then a positive value can be obtained by evaluating *Dist*(*r*_*s*_, *f*_*s*_), a distance between the ground truth *r*_*s*_ and the reference segmentation *f*_*s*_. This value by itself does not reflect something obvious to interpret and we need to compare it to a distribution of values under a null hypothesis of independence between the reference and the automatic segmentation. This distribution can be generated by randomly rotating the maps *g*_*s*_ defined on the sphere, provided that the spherical parameterization *m*_*s*_ preserves areas as much as possible to avoid biases in the spatial distribution of random parcellations on the original mesh $M_{s}$. Eventually the distribution yields a *p*-value.

To obtain random rotations, we generate 3 × 3 matrices with coefficients following independant standard normal distributions, apply a *QR* decomposition, and keep the orthogonal matrices *Q* which are uniformly distributed (Blaser and Fryzlewicz, 2016). It is important to note that our procedure is an improvement of the one in Alexander-Bloch et al. (2017) where 3 angles from uniform distributions along each axes were chosen and introduce a bias as pointed in Blaser and Fryzlewicz (2016).

**Figure 3:**
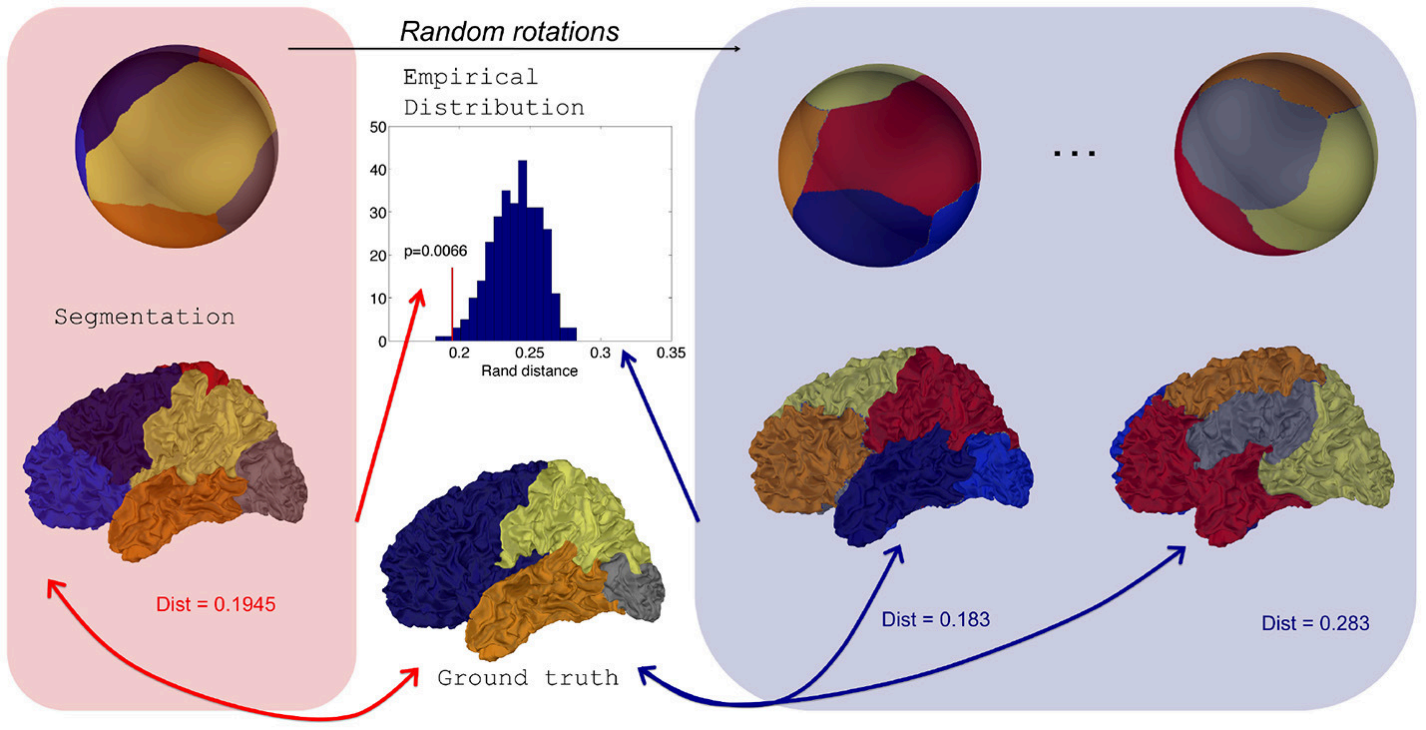
Distribution of Rand distances obtained through random rotations of a parcellation on the sphere. From an initial segmentation projected onto a regularly sampled sphere, random rotations are applied to generate random parcellations onto the sphere and consequently on the original surface. Rand distances are evaluated for each random parcellation which yields an empirical distribution.

We can sum up the procedure in the following algorithm and in Figure 3.

**Algorithm 3:** Significance of Rand Distance

**Require:** Parcellation map labels_a, reference map labels_r spherical mesh sphM, integer Nrot
1: Compute d=Dist(labels_a,labels_r)
2: Initialize rot_d of size Nrot × 1
3: **for** n=1:Nrot **do**
4: Generate a random rotation Q
5: rlabels_a=Interpolate the map labels_a from sphM to Q*sphM
6: rot_d(n)=Dist(rlabels_a,labels_r)
7: **end for**
8: pval= Number of indices n in rot_d such as rot_d(n) < d
9: **return** pval

## 3. Results

### 3.1. Datasets and pre-processings

First we tested our method on a dataset of 62 left hemispheres previously used in Auzias et al. (2013). Triangular meshes were obtained from T1-MRI through the morphologist pipeline of BrainVisa software. Central and Parieto-Occipital sulci were delineated semi-automatically by using Surfpaint (Le Troter et al., 2011). In parallel, Freesurfer was used to obtain (1) a spherical parameterization of each mesh and (2) to generate a parcellation of each surface in 35 regions based on the atlas of Desikan et al. (2006). The 35 regions were then merged in 4 lobes (frontal, parietal, temporal, occipital), insula and cingulate pole following the lobe mapping proposed on Freesurfer wiki^1^.

We also tested the constrained spectral clustering on a dataset of 15 fetuses previously used in Lefèvre et al. (2015). The gestational age ranges from 21 weeks to 34 weeks. T2-weighted images were acquired on axial, coronal, and sagittal planes with a half Fourier acquisition single shot turbo spin echo (HASTE) sequence. Preprocessings consist in high resolution image reconstruction, image segmentation, and mesh generation using an adaptation of BrainVisa processes. Major sulci, when present, were manually traced with Surfpaint.

Next the results are divided in five sections. In sections 3.2, 3.3, 3.4, 3.5 results involve the first dataset. Section 3.6 illustrates the stability of the spectral partitioning with respect to surface deformation and how it can be a powerful tool to parcellate developing brains as revealed by the second dataset.

### 3.2. Descriptive analysis of spectral clusters

**Figure 4:**
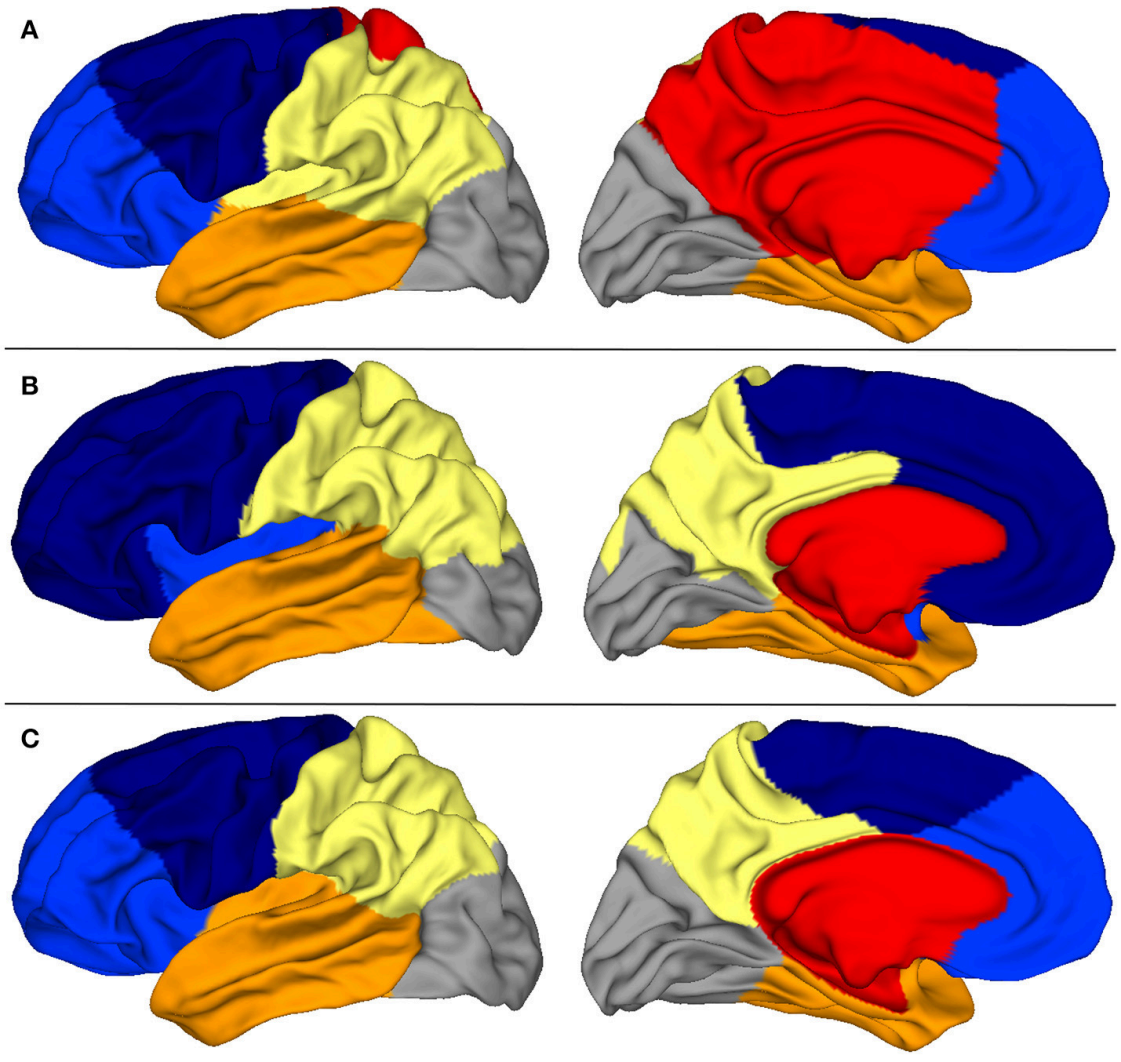
**(A)** Consensus segmentation with the unconstrained spectral approach. **(B)** Consensus segmentation of Freesurfer lobes. **(C)** Consensus segmentation with the constrained spectral approach.

In Figure 4, we show consensus parcellations obtained with the unconstrained (top) and constrained (bottom) approaches (*K* = 6 in both cases) in parallel with a consensus parcellation in lobes obtained with Freesurfer (middle).

The comparison of unconstrained clustering and FreeSurfer reference (first two rows) showed that spectral clusters were strongly reminiscent of anatomical lobes, even with a plain unrefined strategy. Indeed there were several differences between segmentations: (a) the insular lobe was not segmented and was divided by frontal, parietal and temporal lobes, (b) the frontal lobe was divided in two parts (light and dark blue) suggestive of prefrontal and precentral subdivisions, (c) the cingulate pole was poorly delimited and even included in a large mesial region (in red) which extended on a short portion of the external face. This parcel had little anatomical correlate and clearly impaired mesial segmentation. In this view, the introduction of a constrained cingulate pole resulted in several qualitative improvements on the consensus map (third row of Figure 4) that were also present at the individual level in **Figure 6**. By definition of the method, the cingulate pole was perfectly segmented, but it resulted in a global improvement of mesial segmentation, particularly the mesial part of the parietal lobe that was then much more consistent with the one by Freesurfer (second row) while the parietal-occipital boundary remained almost unchanged. Interestingly, the better accordance with Freesurfer segmentation extended on the external face where the temporal-occipital boundary was shifted more posteriorly. Other lobes did not show any important changes.

**Figure 5:**
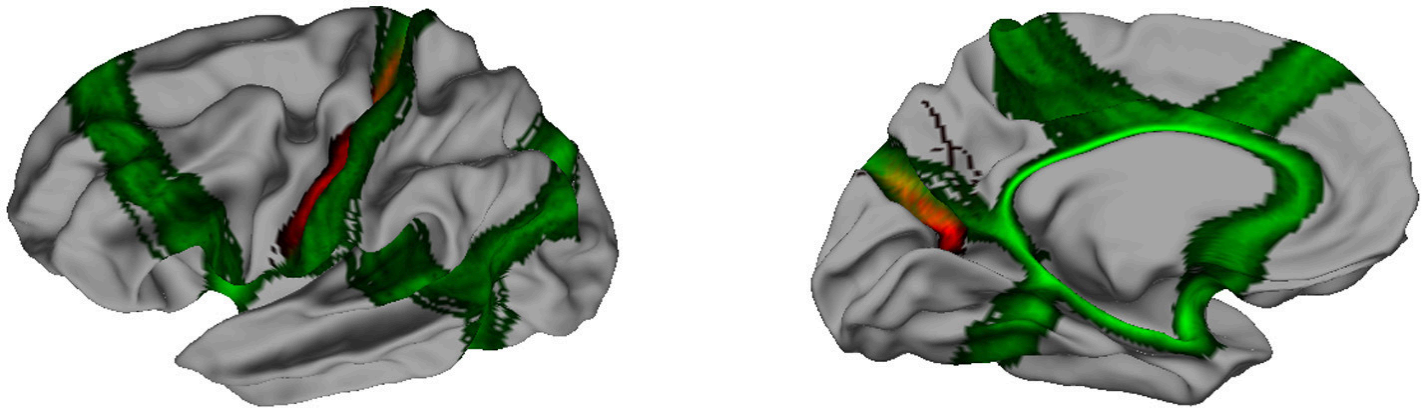
In green, distribution of the clustering boundaries represented on an average brain. In red, distribution of central sulcus **(Left)** and parieto-occipital sulcus **(Right)**. The overlap between the two spatial distribution is encoded by a mix of the two colors.

Striking similarities between spectral segmentation and reference are present on two boundaries, between frontal and parietal lobes (dark blue, yellow), and between parietal and occipital lobes (gray, yellow). Figure 5 illustrates the overlap between the distribution of clustering boundaries (in green) and the distribution of central sulcus and parieto-occipital sulcus (in red), both represented on an average brain obtained with Freesurfer. This figure also offers a first look on the group variability of our approach: All the boundaries are reproducible except an isolated individual variation in the mesial part of the parietal lobe.

### 3.3. Quantitative validation of optimal spectral segmentation

After this visual description, we proposed some quantitative results to emphasize striking similarities between spectral regions and brain lobes. We will now consider mostly the constrained strategy with *K* = 6. In Supplementary Information we added other quantitative results regarding the different clustering strategies (constrained/unconstrained).

#### 3.3.1. Region boundaries and sulci

First we obtained distances between two well-defined boundaries of lobes and corresponding spectral boundaries. Based on our definition 3, we computed two distances *D*_1_ and *D*_2_ for the Central Sulcus (C.S.) and the Parieto-Occipital Sulcus (P.O.S.) of each subject. The numeric values provided a first view on the variability of the segmentations as represented at the group level on Figure 5. In absolute values we obtained a median distance of *D*_1_ = 13.3 mm for the central sulcus and *D*_2_ = 9.2 mm for the parieto-occipital sulcus. Those distances are also compared to the distances obtained with Freesurfer lobes in Table 1. Freesurfer is very accurate on the central sulcus with a median distance of the order of magnitude of the spatial sampling and a bit less for parietal-occipital sulcus that has a more tricky and variable pattern.

**Table 1:** Median distances associated to central sulcus and parieto-occipital sulcus for Spanol and FreeSurfer segmentations.

	**Spanol**	**FreeSurfer**
Central	13.3 mm (2.2–28)	1.4 mm (0.8–2.5)
Parieto-Occipital	9.2 mm (2–27.9)	4.3 mm (1.6–24.6)

When compared to the extremal dimensions of the brain, the median distances with Spanol remain small, with a mean value of 5.7% (from 1% to 11.5%) for the C.S. and 3.9% (from 0.9% to 12.5%) for the P.O.S.

#### 3.3.2. Distances between automatic and freesurfer segmentation

**Figure 6:**
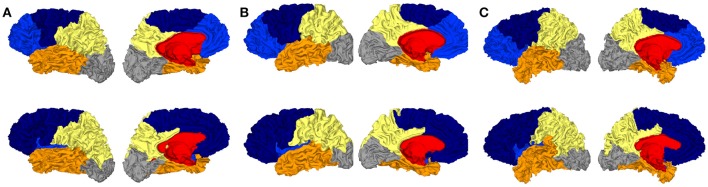
Three spectral segmentatons with constrained cingulate pole (first row) for which the distance with Freesurfer (second row) is the smallest **(A)**, median **(B)**, and the largest **(C)**.

Secondly we evaluated another metric to quantify the adequacy between spectral clusters and anatomical information. The rand distance was computed between each automatic segmentation and the corresponding one obtained by Freesurfer. The values offered another look on the variability of the spectral clusters. Figure 6 shows the segmentations with minimal, median and maximal distances. Quantitatively the values ranged between 0.126 and 0.153 in the constrained case (Figure 6).

#### 3.3.3. Significance of spectral segmentations

To go further we obtained, for each subject, 500 random rotations of Freesurfer lobes on the sphere to generate random partitions that share similar area with the initial Freesurfer segmentation. Those random maps were then used to compute random distances with the different spectral segmentations at the individual level. We then compared the partition distance to the randomly generated distribution of distances. We counted how many subjects among the 62 had a *p*-value below a significance level of 0.01 as obtained from the statistical procedure in 2.5. Constraining the cingulate pole for *K* = 6 improved the ratio of subjects showing a statistical relationship with brain lobes (from 98.4 to 100%).

#### 3.3.4. Optimality regarding the number of clusters

**Figure 7:**
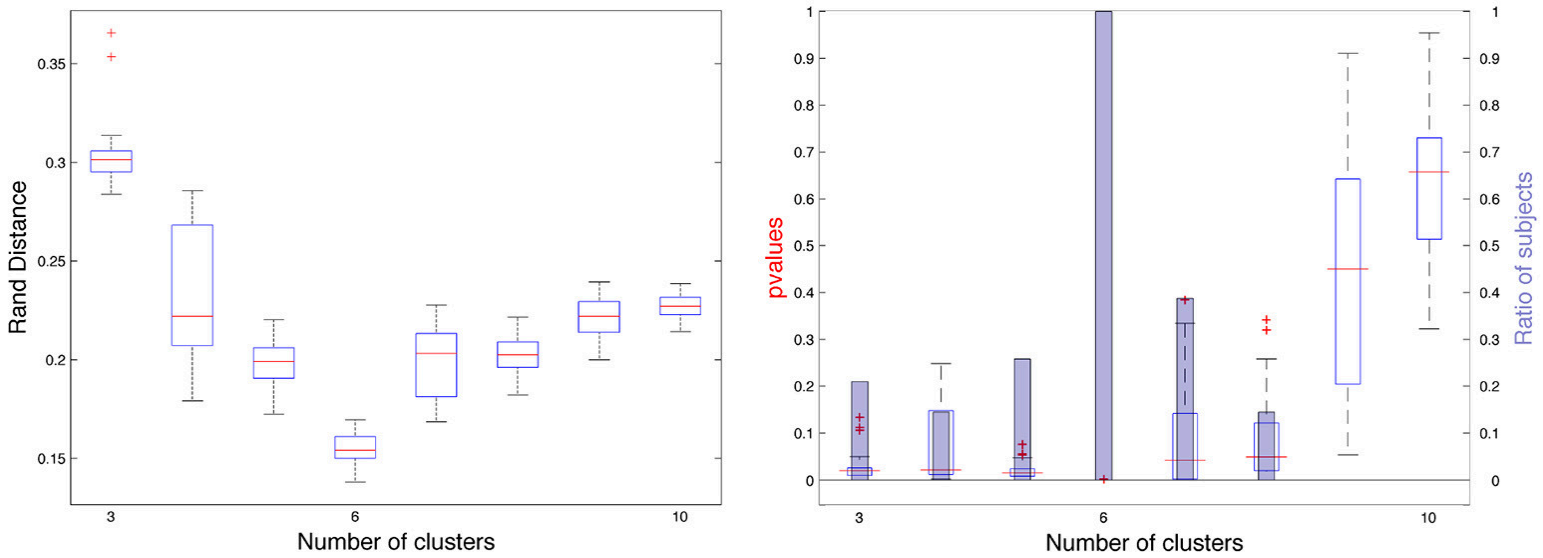
**(Left)** Rand distances between constrained spectral segmentation with *K* regions and Freesurfer segmentation with 6 lobes. **(Right)** *p*-values for each subject represented as boxplots and proportion of subjects (blue bars) for which the *p*-value is < 0.01.

We computed partition distances between constrained spectral approaches with *K* labels and *K* eigenvectors when *K* varies between 3 and 10, given that *K* = 2 is pointless (two regions with the cingulate pole and its complement part). On Figure 7 (left), we observed the distribution of rand distances and the median values (red) ranged between 0.15 and 0.3 with a clear minimum for *K* = 6. On Figure 7 (right), we added information obtained through our random rotations procedure. We displayed both the distribution of individual *p*-values (boxplot) and the ratio of subjects for which the rand distance was significant with respect to the random distribution. This ratio was 1 for *K* = 6 and 0.38 for *K* = 7 (24 subjects). For all the other values, the ratio was below 0.3 suggesting poor association between spectral segmentations and Freesurfer lobes. On Figure 2SI we showed all the consensus segmentation obtained when *K* varied between 3 and 10.

### 3.4. Group analysis of spectral clustering

In this part we further analyzed the ability of the spectral clustering approaches to delineate automatically brain lobes. We evaluated first the overlaps between each Freesurfer lobe and the corresponding spectral region and then by comparing the two strategies described in section 2.3. Those steps necessitated before a consistent matching between the regions, at the subject and group level, by using the hungarian algorithm.

#### 3.4.1. Lobe by lobe statistics

Beyond the global rand distances shown in S.I., we computed Dice coefficients between each Freesurfer lobe and the corresponding one in two spectral approaches (unconstrained, constrained, with 6 eigenvectors). The corresponding frontal lobe was obtained by concatenating two spectral regions (light and dark blue in most of the previous figures). The Insula was not included since it had no corresponding region after the grouping strategy for the frontal lobe. The results were summarized in Table 2. For all lobes except for the occipital one (almost no change), the mean Dice coefficients were improved by constraining the cingulate pole, particularly for the parietal lobe.

**Table 2:** Dice coefficients between Freesurfer lobes and each of the 5 corresponding regions obtained with the spectral segmentation.

**Cingulate**	**Frontal**	**Parietal**	**Temporal**	**Occipital**
0.39 ± 0.04	0.89 ± 0.01	0.66 ± 0.05	0.82 ± 0.02	**0.82 ± 0.03**
**0.91 ± 0.02**	**0.94 ± 0.01**	**0.83 ± 0.03**	**0.87 ± 0.01**	0.81 ± 0.03

*Note that the corresponding frontal lobe is obtained by concatenating two spectral regions. The Insula is not included since it has no corresponding region after the grouping strategy in frontal lobe. First row: unconstrained method with 6 eigenvectors. Second row: constrained method with 6 eigenvectors. The bold value indicate the largest value between the two rows*.

#### 3.4.2. Comparison of the two group strategies

In Table 3 we showed the 6 Dice coefficients between the regions obtained with the individual spectral clustering and the joint group clustering. We remarked that dice values were large (above 0.93) and close to the perfect overlap (= 1). For all lobes except the temporal one, the overlap was improved when the constraint on the cingular pole was added.

**Table 3:** Dice coefficients for each of the 6 spectral regions obtained with the individual and the group strategy.

**Front**	**PreFront**	**Occip**	**Pariet**	**Tempo**	**Mesial**
0.95 ± 0.03	0.93 ± 0.04	0.96 ± 0.03	0.94 ± 0.03	**0.97 ± 0.02**	0.97 ± 0.02
**0.96 ± 0.02**	**0.98 ± 0.01**	**0.97 ± 0.02**	0.94 ± 0.03	0.96 ± 0.03	**1.00 ± 0.00**

*First row: unconstrained method with 6 eigenvectors. Second row: constrained method with 6 eigenvectors. The bold value indicate the largest value between the two rows*.

For the subject with minimal overlap the two lowest Dice coefficients were 0.88 for both temporal and parietal lobe (see SI for the visual comparison).

### 3.5. Features of the spectral clustering

**Figure 8:**
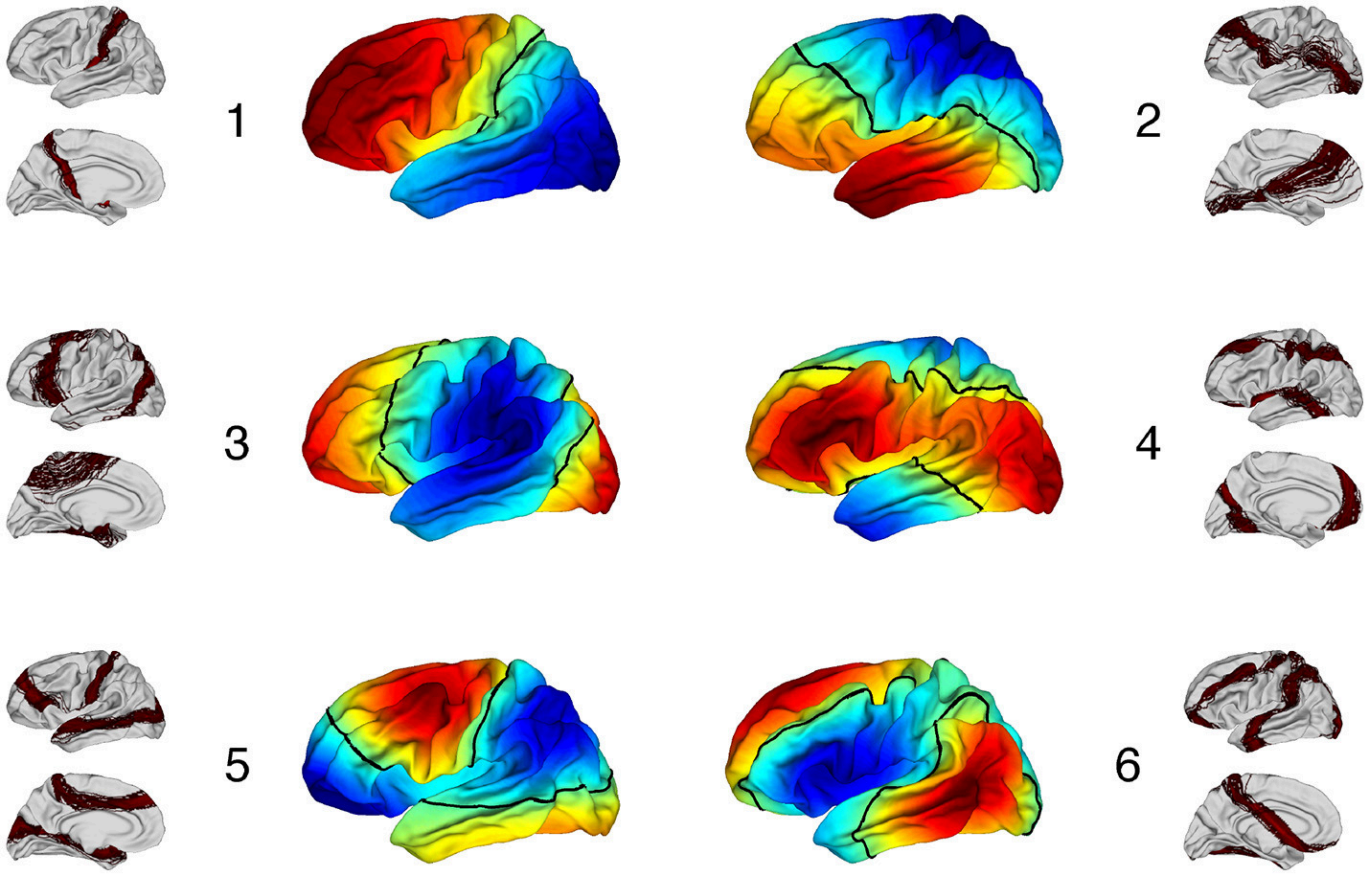
Center: Mean pattern of the 6 first non trivial eigenfunctions. Positive values are in red, negative values in dark blue and zero values in green with the nodal line in black. Around each eigenfunction is represented the variability of nodal lines across the 62 subjects.

For each subject, the 6 first non trivial eigenfunctions were projected onto a brain template by using Freesurfer. On Figure 8 we displayed the mean pattern of each eigenvector used in the spectral clustering. For each eigenfunction the variability is illustrated by showing the nodal lines across the 62 subjects.

It is remarkable to observe that the individual nodal lines are distributed along consistant directions. The variability of those nodal lines is simply represented by a shift transverse to the main direction. In a dual perpective, one observes for each eigenfunction a clear oscillating pattern with several connected regions sharing the same sign (so called nodal domains). For each subject, there are respectively 2, 2, 2, 2, 3, 3 nodal domains for the 6 first eigenfunctions in increasing order. Those results illustrate the perfect stability of eigenfunction from a topological point of view.

### 3.6. Robustness of the constrained spectral segmentation

#### 3.6.1. Effect of surface smoothing

**Figure 9:**
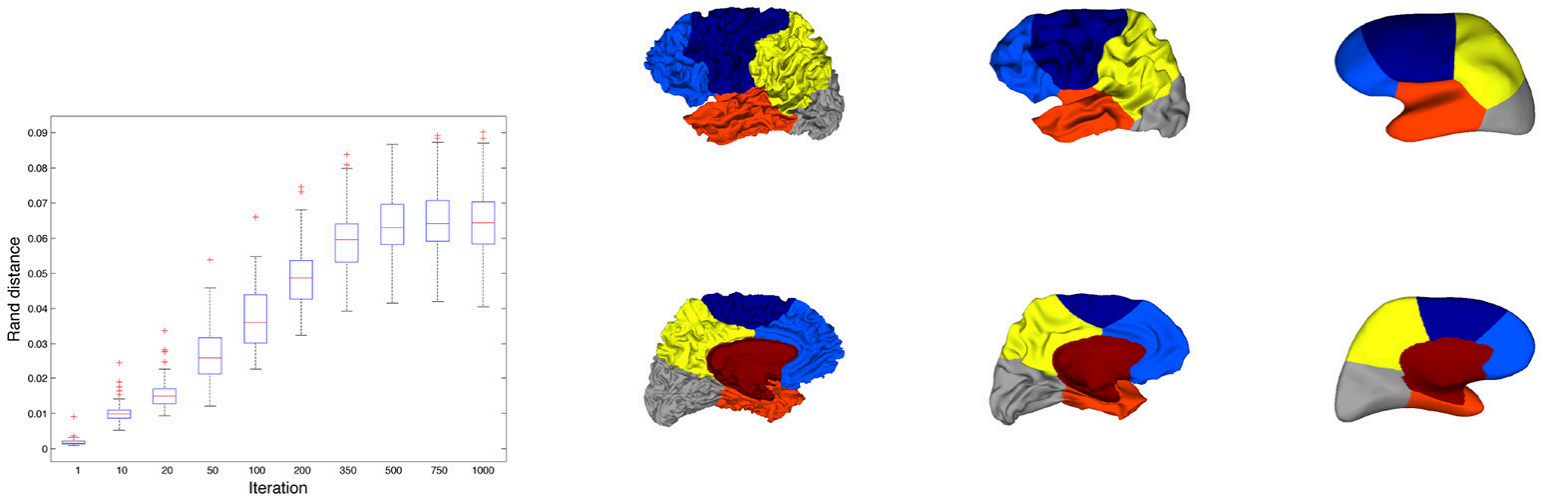
**(Left)** Distribution of rand distances between initial constrained segmentations and segmentations for increasing number of iterations in the smoothing process. **(Right)** Illustration of the smoothing process for a surface whose rand distance correspond to the median value (0.06) of the distance distributions for 1,000 iterations. 3 iterations are considered (0, 100, and 1,000).

For each subject we applied the mean curvature flow to smooth the folded geometry of the cortical meshes. For several iterations of the process (*t* = 1, 10, 20, 50, 100, 200, 300, 500, 750, 1, 000) we ran the constrained spectral clustering procedure with 6 eigenvectors and computed the partition distance between the resulting segmentation and the initial segmentation. The variability of this distance was illustrated in Figure 9 (left). We observed a regular increase of the mean value till an interval [0.04−0.09] and a spreading of the distribution as well. Compared to the rand distances found between constrained spectral parcellations and Freesurfer lobes (around 0.15 on Figure 7 or Table 2SI) which reflected good agreements, one can therefore consider that the perturbation of spectral parcels during the smoothing process is small.

In Figure 9 (right) we observed the constrained spectral clustering for a surface whose rand distance correspond to the median value (0.06) of the distance distributions for 1000 iterations. The most visible change can be seen on the mesial face with a slight bifurcation of the yellow/blue boundary that has a counterpart on the external face close to the central sulcus.

#### 3.6.2. Segmentation of the immature brain

**Figure 10:**
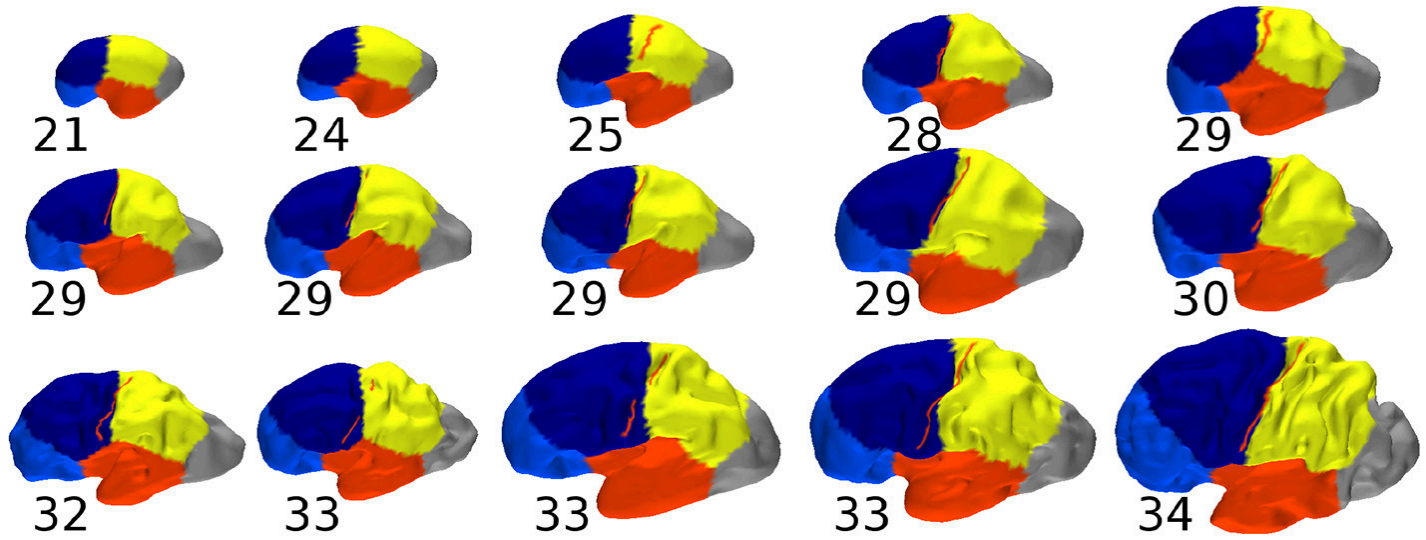
Constrained spectral segmentations of fetal brains with preserved proportions and gestational age in weeks. The central sulcus, when present, is superimposed in red.

In Figure 10 we showed the constrained spectral segmentation during early development on 15 fetuses whose gestational age ranges from 21 weeks to 34 weeks. We observed an informative consistency between the different regions across ages. Moreover the position of the parcells was qualitatively similar to what was observed for adult brains. But more precisely the analogous of frontal/parietal boundary crossed the central sulcus which was not the case for adult brains as revealed in particular by Figure 5. It is also possible to quantify the distance between C.S. (respectively P.O.S) and its corresponding boundary, except for the two youngest subjects (21 and 24 weeks gestational age). For the C.S. the absolute values range from 0.7 mm (28 w GA) to 6.3 mm (25 w GA) while for the P.O.S it was from 1.3 mm (25 w GA) to 6.1 mm (33 w GA). Regarding the percentage of the largest distance, the mean value is 2.1% (from 0.9 to 8.1%) for C.S., and 3.44% (from 0.0 to 6.2%) for the P.O.S. In both cases the correlation of those values with age were found non significant (*R* = 0.46 and *R* = −0.07 respectively) when correcting for the increasing global size of the cortical surface, as measured by its longest geodesic (Lefevre et al., 2012).

## 4. Discussion and conclusion

The major applicative result of our general Spanol approach is the possibility to segment a cortical surface in a few parcels that have strong similarities with brain lobes. These similarities can be precisely quantified by partition distances and a dedicated statistical procedure, which has been proposed to determine how far the resulting parcels stood from randomly segmented lobe-sized parcels. The segmentation process can be entirely unsupervised, using only the geometrical properties of the hemispheric mesh shape. However, we improved dramatically the global overlap with the four main lobes (frontal, parietal, temporal and occipital) obtained by Freesurfer software by adding limited constraints only, excluding points that belong to non-cortical regions of the mesh, namely the cingulate pole. This could be seen as a semi-supervised clustering at the level of the whole mesh, but remains strictly unsupervised regarding the cortical level. We would insist on two main neurological relevancies of Spanol, and further discuss interesting methodological considerations raised by its development.

### 4.1. Practical aspects

First, to our knowledge it is the first attempt to extract in a fast, simple, reproducible, and almost unsupervised manner a parcellation of a cortical surface in lobes. This approach does not require any atlas and can be applied as it is to triangular meshes, at the individual level, in a purely intrinsic way. The computation time is limited to a few seconds for each surface.

Our unsupervised spectral clustering approach identifies boundaries that may be less accurate for C.S. and P.O.S than the ones obtained by FreeSurfer in a pure supervised manner. The interpretation of the larger discrepancy with our spectral approach clearly depends on the expectancies one can have for a new totally unsupervised approach by comparison to a long-optimized multi-input one. The main difference is on the central boundary, for which the spectral approach generates a kind of global posterior shifting, even if the limit can be really accurate in some subject. This interesting point requires further investigation that we are currently conducting.

Although some local improvements can be expected according to experimental needs, they may be achieved with complementary segmentation steps, either supervised or not, following Spanol backbone. For instance, the strict respect of bottom sulci lines along some lobar boundaries (e.g., for C.S. or P.O.S) would be a possible finishing stage, in the same spirit as Klein and Tourville (2012). It could be also interesting to consider this segmentation problem in a semi-supervised manner following for instance (Lu and Carreira-Perpinán, 2008). The exclusion of otherwise segmented Insula before spectral clustering has been also tested without disturbing the relevance of lobar boundaries. Indeed, the implementation of Spanol could allow regional analysis of morphometric parameters, in the spirit of Toro et al. (2009), to be rapidly performed without requiring complicated pipelines and potentially skewing normalization steps.

### 4.2. Link with neurodevelopmental questions

Second and more fundamental, spectral lobes are obtained from the intrinsic geometrical informations contained in the low-frequency eigenfunctions only. Yet, the lobar limits given by Spanol are not only consistent with the classical sulcal ones (C.S., P.O.S) but also for the ill-defined one such as the lateral parietal occipital or the temporal occipital. Even the segmentation of a prefrontal sub-lobar region seems rather consistent. While classical anatomy relies on almost *ad hoc* landmarks such as the preoccipital notch, or locally defined virtual lines (e.g., occipital temporal limit), spectral analysis kind of intergrade the whole shape into objectively defined boundaries. Indeed, the fact that lobes can be intrinsically defined from the shape of the brain is *per se* an intriguing result. Interestingly, not all the geometrical information is required, but only the one of the low-frequency eigenfunctions that give mostly information on the global shape of the brain (Seo and Chung, 2011; Germanaud et al., 2012). It may suggest a strong relationship between global shape of the cortical surface and the appearance of great primary sulci that defined main lobar limits (C.S., P.O.S), but also with functional aspects associated with lobes. Anyway, this dependency upon global shape only would explain the stability of spectral segmentation of lobes along development regardless of the full expansion of the main sulci. This claim has been empirically verified by smoothing the cortical surfaces to decrease the influence of sulci and gyri in the geometry and by quantifying the stability of spectral lobes during this deformation.

Moreover the good reproducibility of spectral lobes on a small population of fetuses with various folding complexity is another argument in favor of a very early determination of brain lobes, even from a very smooth cortical surface. This question is also closely intertwined with the emergence of primary folds during fetal development. Among various explanations of this phenomenon, the mecanical hypothesis (Tallinen et al., 2016) is seducing in our case because of oscillatory properties of the surface as proxies of its ability to fold and determine the position of primary folds. Indeed, the question of the relationship between shape and function is all the more interesting since one inverts the classical paradigma and addresses the fact that the determinent of shape may impact the functional outcome (Foubet and Toro, 2015). So far, it is a very general question, not restricted to the lobar one.

From a more practical point of view, Spanol offers a promising way to define automatically brain lobes in fetuses or newborns because of this nice continuity in the ontogeny. It would remain to test more systematically our method in larger longitudinal databases with complementary information provided by cytoarchitecture, connectivity or functions.

### 4.3. Final remarks

Spanol provides a segmentation of cortical surface by using a *K*-means clustering of Laplace-Beltrami operator eigenfunctions. In other terms, only few low frequency descriptors of the brain geometry are required (optimum with 6 in our work) to provide a given number of relevant connected regions on the cortical surface. This method has been used at the individual level and at the group level by concatenating all the eigenfunctions of each subject of the group. A major advantage of the second approach is the ability to obtain directly a consistent labeling of the resulting parcels. In the individual case, it requires to solve the assignment problem to match parcels of different subjects. From a computational point of view the group spectral clustering with 62 meshes of 100−150*k* vertices takes approximately 1 min on a standard laptop which is comparable to 62 individual *K* means (*K* = 6 in our case). The results showed strong similarities between the segmentations obtained at the individual or group level as measured by the rand distance. This result legitimates the group spectral clustering on our data as a way to segment individual meshes in a consistent, reliable and fast manner. It also suggests a very reproducible pattern in the low frequency eigenfunctions of cortical surfaces which has already been pointed out in several works (Germanaud et al., 2012; Lombaert et al., 2013b; Lefèvre and Auzias, 2015). More precisely in Lombaert et al. (2013a) the authors observe that between 5 and 15 eigenvectors perform equally well in terms of brain regions overlap when used in their spectral registration algorithm. Besides it could be interesting to explore whether a group spectral clustering procedure like our's could be applied successfully to a larger number of regions. Indeed, in the field of computer graphics the co-segmentations of shapes with spectral approaches have been shown efficient but often tested for limited number of components (Sidi et al., 2011). Conversely studies with other kinds of neuroimaging data (fMRI) suggested that spectral clustering approaches would not be satisfactory for large values of *K* (Thirion et al., 2014).

A novelty in our approach concerns the statistical procedure to test whether a given brain segmentation could be considered as randomly sampled from a null distribution of brain parcellations sharing similar properties. We escaped the intractable (and biologically irrealistic) combinatoric of testing all possible partitions of a triangular mesh by proposing random rotations of a segmentation map onto a reference sphere and computing distances with a reference segmentation. This approach is able to find statistical associations between spatial partitions independently of the partition distance used and of the disparity between the number of clusters in the reference and the automatic segmentations. This procedure takes approximately a few minutes for each subject for 500 random samples. With this statistical tests, we are able to determine which subjects have a spectral parcellation that is significantly close to a reference one. At the end we can find the number of clusters which maximizes the number of significant subjects.

Regarding the supervised information injected in fixing the cingulate pole, we have adopted a strategy where eigenfunctions of the original surface are used in the spectral clustering while excluding the points of the constraint. Something more mathematically natural would have been to consider the eigenfunctions of the Laplace-Beltrami Operator with boundary conditions on the borders of the cingular pole. But our choice was motivated by purely pragmatic considerations regarding unsatisfactory segmentations in the second case. Why the sound mathematical framework of Neuman or Dirichlet boundary conditions has failed and why our *ad-hoc* treatment of boundaries in the cingulate pole is more successful, remains an interesting question that might even be more general than in our specific context of neuroanatomy.

As closing remarks, our work illustrates, from the applicative point of view of brain anatomy, one of the fascinating properties of the Laplace-Beltrami Operator. For a long time low-frequency patterns of its first eigenfunctions have been well described by theorems like Courant's nodal one, which estimates the number of spatial oscillations. Despite a few theoretical results, spectral clustering remains a rather empirical way to combine the geometrical information contained in the eigenfunctions. Still, our results show its ability to unveil structure in datasets such as those in neuroimaging. Besides we rediscovered after (Jin et al., 2005) that varying slightly the number of eigenvectors can improve clustering results. This discrepancy with the idealized view of spectral clustering offers also stimulating theoretical perspectives in the future.

## Ethics statement

All subjects gave written informed consent in accordance with the Declaration of Helsinki. The protocol was approved by the Ethical Commitee of Aix-Marseille University.

## Author contributions

JL and DG: designed research; JL, AP, JM: conducted analyses; FD, NG: provided and processed fetal data; JL, GA, and DG: wrote the paper. All authors took part in the scientific discussion at multiple stages of the study.

### Conflict of interest statement

The authors declare that the research was conducted in the absence of any commercial or financial relationships that could be construed as a potential conflict of interest.

## Footnotes

^1^Cortical Parcellation/Lobe Mapping: https://surfer.nmr.mgh.harvard.edu/fswiki/CorticalParcellation
