# Supplementary material for: SPANOL (SPectral ANalysis of Lobes): A Spectral Clustering Framework for Individual and Group Parcellation of Cortical Surfaces in Lobes
Source: Front Neurosci. 2018 May 31;12:354. doi: 10.3389/fnins.2018.00354 (PMC5990633; doi:10.3389/fnins.2018.00354)
Supplement: Supplementary file 1 [file Presentation_1.pdf]

Supplementary Information to ”Spectral Analysis of lobes: A spectral clustering framework for group parcellation of cortical surfaces in lobes.

Variability of unconstrained segmentations On Figure SI we showed three segmentations representing the variability of the unconstrained spectral method with $K = 6$ following the rand distance.

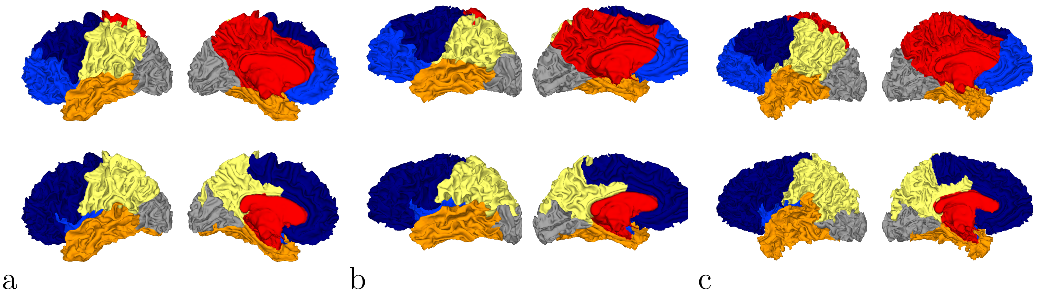

a b c

Figure 1: Three unconstrained spectral segmentations (first row) for which the distance with Freesurfer (second row) is the smallest (a), median (b) and the largest (c).

Rand distances Mean results were summarized in Table 2. By using the rand metric the constrained method with 6 eigenvectors yielded the minimal distances followed by the unconstrained one with 6 eigenvectors.

Consensus parcellations for varying K

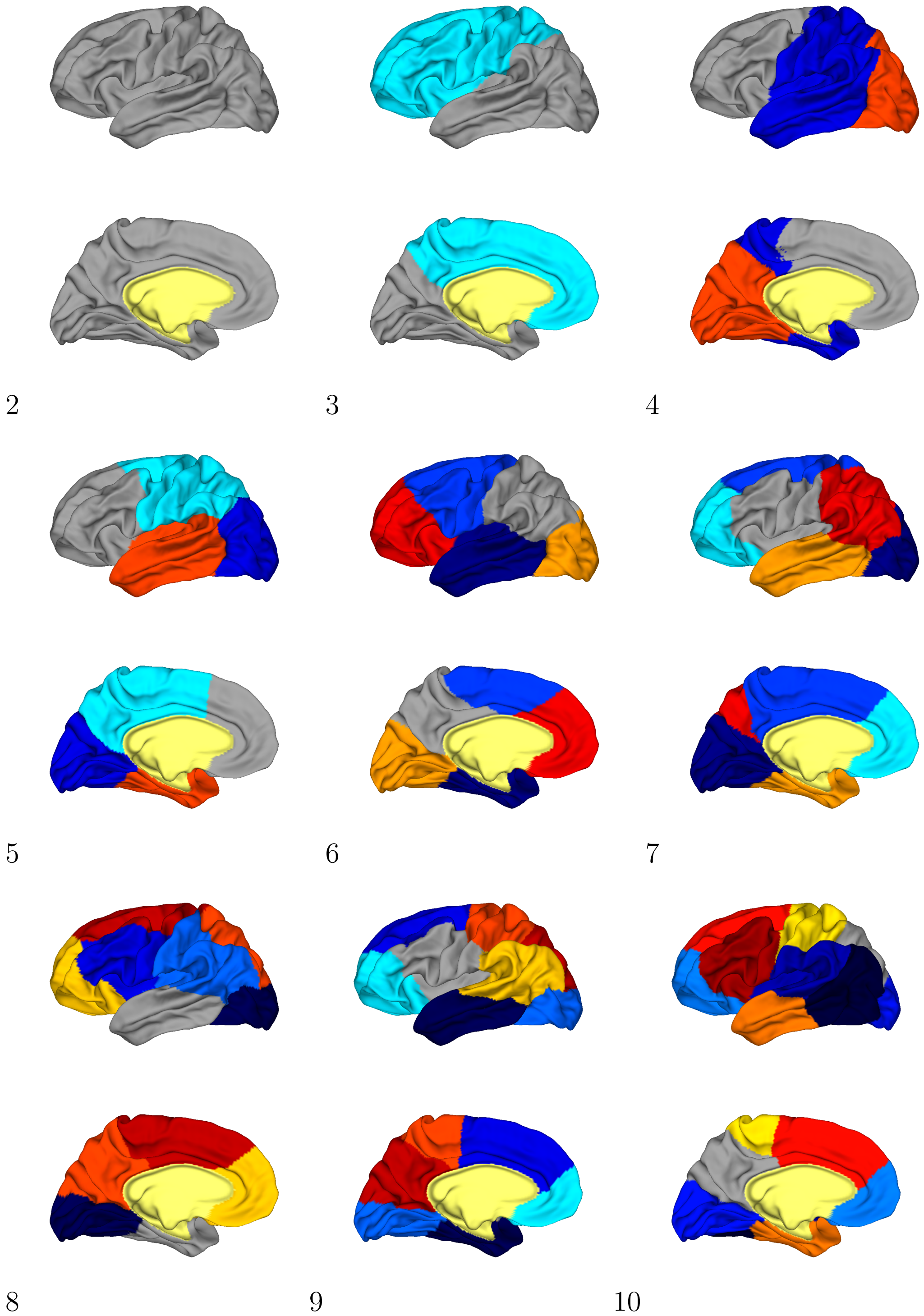

Figure 2: From left to right, consensus parcellations for spectral segmentations with K regions (from 2 to 10).

	Unconstrained		Constrained	
	6 ev	7 ev	5 ev	6 ev
Individual	0.186±0.006	0.199±0.015	0.190±0.007	0.140±0.006
Group	0.186±0.010	0.200±0.008	0.190±0.008	0.141±0.008

Table 1: Mean rand distances between automatic and freesurfer segmentations. Each column corresponds to a specific strategy in the spectral clustering (with or without constraints in the cingular pole), K or $K-1$ eigenvectors used. In each cell we report the mean distance across a group of 62 subjects.
